# Novelties in the genus *Viridantha* Espejo (Tillandsioideae, Bromeliaceae)

**DOI:** 10.3897/phytokeys.132.36959

**Published:** 2019-10-02

**Authors:** Rodrigo Alejandro Hernández-Cárdenas, Alejandra Serrato Díaz, Ana Rosa López-Ferrari, Adolfo Espejo-Serna

**Affiliations:** 1 Herbario Metropolitano, Departamento de Biología, División de Ciencias Biológicas y de la Salud, Universidad Autónoma Metropolitana Unidad Iztapalapa, C.P. 09340, Ciudad de México, México Universidad Autónoma Metropolitana Mexico Mexico; 2 Laboratorio Divisional de Biología Molecular, División de Ciencias Biológicas y de la Salud, Universidad Autónoma Metropolitana Unidad Iztapalapa, C.P. 09340, Ciudad de México, México Universidad Autónoma Metropolitana Federal District Mexico

**Keywords:** Hidalgo, México, Oaxaca, *
Tillandsia
*, *
Viridantha
*

## Abstract

Based on morphological evidence, we propose to raise Tillandsia mauryana forma secundifolia to species level with the name *Viridantha secundifolia* (Ehlers) Hern.-Cárdenas, Espejo & López-Ferr. *Viridantha secundifolia* can be readily distinguished by the falciform rosettes, the broadly oblong to square, 1–1.2 × 0.8–1.1 cm leaf sheaths and by the 1.8–2 × 0.7–1.2 cm floral bracts. Additionally, we describe and illustrate *Viridantha uniflora* Hern.-Cárdenas, Espejo & López-Ferr., from the state of Oaxaca, Mexico. The new species is morphologically similar to *Viridantha boqueronensis*, but differs by the nearly square leaf sheaths, 1.3–1.5 × 0.4–0.5 cm spikes and by the presence of only one flower per spike. A key to the taxa, morphological descriptions, list of specimens examined, illustrations and a distribution map of the described taxa are included.

## Abstract (es)

Con base en evidencia morfológica, proponemos elevar a nivel de especie a Tillandsia mauryana forma secundifolia con el nombre *Viridantha secundifolia* (Ehlers) Hern.-Cárdenas, Espejo & López-Ferr. *Viridantha secundifolia* puede distinguirse fácilmente por las rosetas falciformes, las vainas foliares ampliamente oblongas a cuadradas, de 1–1.2 × 0.8–1.1 cm y por las brácteas florales de 1.8–2 × 0.7–1.2 cm. Además, se describe e ilustra *Viridantha uniflora* Hern.-Cárdenas, Espejo & López-Ferr., del estado de Oaxaca, México. La nueva especie es morfológicamente similar a *Viridantha boqueronensis* pero difiere por las vainas foliares cuadradas, la espigas de 1.3–1.5 × 0.4–0.5 cm y por presentar una sola flor por espiga. Se proporciona una clave de identificación, descripciones morfológicas, lista de ejemplares examinados, ilustraciones y un mapa de distribución de los taxa descritos.

## Introduction

The genus *Viridantha* Espejo (Tillandsioideae, Bromeliaceae) is endemic to Mexico and includes 14 species (Hernández-Cárdenas et al. 2018). Due to its morphological characteristics, Smith and Downs (1977) classified some species, now placed in *Viridantha*, in Tillandsia subgenus Allardtia [*V. atroviridipetala* (Matuda) Espejo, *V. ignesiae* (Mez) Espejo, *V. mauryana* (L.B. Sm.) Espejo and *V. plumosa* (Baker) Espejo] and others in Tillandsia subgenus Tillandsia [*V. ehrenbergii* (= *V. tortilis* (Klotzsch ex Baker) Espejo) and *V. lepidosepala* (L.B. Sm.) Espejo]. Gardner (1986) included *Viridantha* in Tillandsia subgenus Tillandsia and Till (2000) included it in Tillandsia subgenus Allardtia, but as a separate group from the rest of the species in that subgenus. Espejo-Serna (2002), based on morphological characteristics, considered that this group of species constituted a distinct genus from *Tillandsia* L. and called it *Viridantha*.

*Viridantha* species are herbaceous plants with leaves arranged in acaulescent rosettes; protandrous flowers with petals dark green towards the apex and white towards the base; stamens equal in length, included, with filiform filaments and sub-basifixed anthers and simple-erect type style branches (Espejo-Serna 2002). The last taxonomic revision for Tillandsioideae subfamily, based on multi-loci DNA sequences phylogeny, proposed to circumscribe *Viridantha* and the *Tillandsia tectorum* E. Morren complex as Tillandsia subgenus Viridantha (Espejo) W. Till & Barfuss (Barfuss et al. 2016). However, some of the systematic changes proposed by Barfuss et al. (2016) were not supported by molecular and morphological data (Gomes-da-Silva and Souza-Chies 2017).

It should be mentioned that, in all the phylogenies reconstructed so far, *Tillandsia* emerged as polyphyletic (Gardner 1986; Terry et al. 1997; Horres et al. 2000; Barfuss et al. 2004, 2005; Barfuss 2012; Barfuss et al. 2016; Gomes-da-Silva and Souza-Chies 2017) or paraphyletic (Terry and Brown 1996; Benzing et al. 2000; Givnish et al. 2007; Donadío et al. 2015) and, consequently, its validity as a formal taxonomic entity (genus) is unacceptable. On the other hand, *Viridantha* has always been monophyletic and related to the *Tillandsia tectorum* complex (Barfuss et al. 2004, 2005, 2016; Barfuss 2012). The members of *Viridantha* have morphological, ecological and geographical coherence, all the species are easily recognised by their vegetative and reproductive characteristics and can be distinguished from the rest of the species of *Tillandsias. l.* Furthermore, *Viridantha* species are mostly saxicolous and all are endemic to Mexico. Moreover, the species of the *Tillandsia tectorum* complex are clearly distinct from *Viridantha* by the presence of caulescent rosettes and petals purple towards the apex and white towards the base, besides the taxa of *T. tectorum* clade being endemic to northern Peru and southern Ecuador (Hromadnik 2005). By the above mentioned reasons, we maintain *Viridantha* as a genus. It becomes necessary to carry out more studies using a larger number of species and/or characteristics to propose a more precise and objective classification of *Tillandsias.l.* and their relatives.

As a result of botanical explorations for the project Phylogeny of the genus *Viridantha* Espejo (Tillandsioideae; Bromeliaceae), we collected specimens of two different populations of *Viridantha*: the first one in the vicinity of Tolantongo, in the municipality of Metztitlán, state of Hidalgo, corresponds to Tillandsia mauryana L. B. Sm. forma secundifolia Ehlers. This form can be readily distinguished from the typical form by the falciform rosettes, the broadly oblong to square, 1–1.2 × 0.8–1.1 cm leaf sheaths, by the 1.8–2 × 0.7–1.2 cm floral bracts and other morphological characters, so we propose to raise it to species level. The second one comes from the municipality of Santos Reyes Tepejillo, in the state of Oaxaca. Initially, we thought that these specimens could correspond to *Viridantha boqueronensis* (Ehlers) Hern.-Cárdenas, Espejo & López-Ferr.; however, after a careful and detailed revision of living and herbarium specimens, including types, we conclude that these populations correspond to an undescribed taxon.

## Materials and methods

Plants were collected in Hidalgo and Oaxaca, Mexico. The material was dried and measurements and descriptions were prepared from herbarium specimens. The vouchers were deposited in UAMIZ. The morphological terms used in the descriptions were based on Radford et al. (1974) and Scharf and Gouda (2008). We revised herbarium material deposited at CHAPA, FCME, GH, IBUG, IEB, MEXU, UAMIZ and WU and all specimens are cited in the text or in Appendix 1. Comparison of the new species with *Viridantha boqueronensis*, *V. mauryana* (L.B. Sm.) Espejo and *V. penascoensis* (Ehlers & Lautner) Espejo & López-Ferr. and other morphologically related taxa was based on the protologues, living specimens collected at the type localities, as well on herbarium specimens (Appendix 1). The herbarium acronyms followed Thiers (cont. updated).

## Results

### Viridantha secundifolia

Taxon classificationPlantaePoalesBromeliaceae

(Ehlers) Hern.-Cárdenas, Espejo & López-Ferr., comb. et stat. nov.

E2D60E6C-E656-552D-97BE-048AF7CD5CCF

urn:lsid:ipni.org:names:60479388-2

#### Basionym.

Tillandsia mauryana L.B. Sm. forma secundifolia Ehlers, Die Bromelie. Sonderheft 6: 56–60. Figs pp. 56, 57, 60. 2009.

#### Type.

MEXICO. Hidalgo: Metztitlán, 1300 m a.s.l., 12 February 1992, *J. Lautner L92/3* (holotype: MEXU not found); Hidalgo, prope Tolantongo “Tolontogo”, 1900 m a.s.l., 22 February 2006, *R. Ehlers & M. Kretz EM061802* (paratype: WU not found). Lectotype (here designated): figure page 56, Die Bromelie. Sonderheft 6: 56-60. 2009.

#### Description.

Plants saxicolous, flowering 10–13 cm tall, 12–14 cm diameter; rosettes acaulescent, solitary or caespitose, falcate in outline. Leaves numerous, longer than the inflorescence; sheaths pale brown on both surfaces, broadly oblong to nearly square, 1–1.2 cm long, 0.8–1.1 cm wide, glabrous towards the base on both surfaces; blades falcate, densely white-greyish lepidote, narrowly triangular, 4.5–7 cm long, 0.5–0.8 cm wide, apical portion long attenuate. Inflorescence short pedunculated, falcate, one-branched, with 3–5 spikes; peduncle 1–1.5 cm long, 3–5 mm diameter, covered by the peduncle bracts; peduncle bracts similar to the leaves but reducing in size towards the apical portion, densely white-greyish lepidote; spikes reddish-rose, erect and appressed, flattened, elliptic, 2.5–3.5 cm long, 1–1.5 cm wide; flowers distichous, erect and appressed, 3–5 by spike; floral bracts reddish-rose at the apex, yellowish-green towards the base, ovate, 1.8–2 cm long, 0.7–1.2 cm wide, apex acute to acuminate, ecarinate to slightly carinate at the apex, lepidote abaxially; sepals pale green, lanceolate, 1.2–1.5 cm long, 0.3–0.4 cm wide, apex acute, the two adaxial ones carinate, lepidote abaxially; petals dark green, narrowly oblong, 2–2.5 cm long, 0.25–0.3 cm wide, apex rounded to obtuse; filaments white, 1.4–1.6 cm long; anthers pale to dark green, 2.5–3 mm long; ovary broadly ovoid, 3.5–4.5 mm long, 2.5–3.5 mm diameter; style white, 6–10 mm long, included; style branches green. Capsules 1.5–1.8 cm long, 5–8 mm diameter; seeds fusiform, 3–4 mm long, coma 0.8–1.2 cm long.

#### Habitat and ecology.

**Figure 1.**
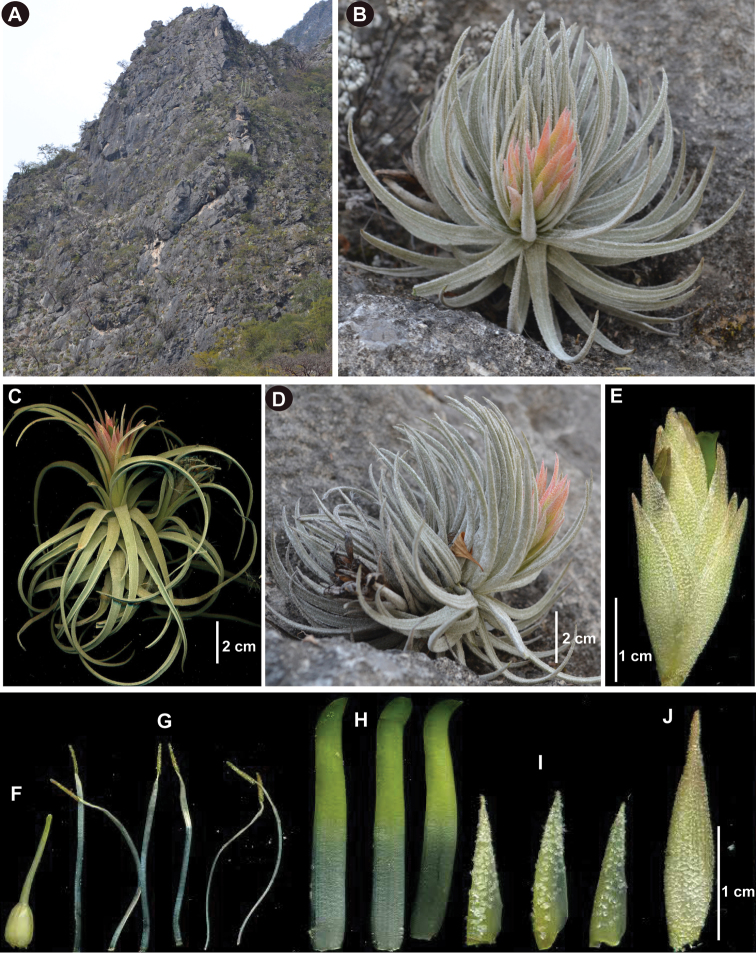
Morphological comparison between *Viridantha mauryana* and *V. secundifolia* (Ehlers) Hern.-Cárdenas, Espejo & López-Ferr. *V. secundifolia***A** habit **B, D** plant with inflorescence **E** spike **F** pistil **G** stamens **H** petals **I** sepals **J** floral bract (voucher: *Hernández-Cárdenas and Sarabia 2136*, UAMIZ). *V. mauryana***C** plant with inflorescence (voucher: *Hernández-Cárdenas et al. 2090*, UAMIZ). Photographs by R. Hernández-Cárdenas.

**Figure 2.**
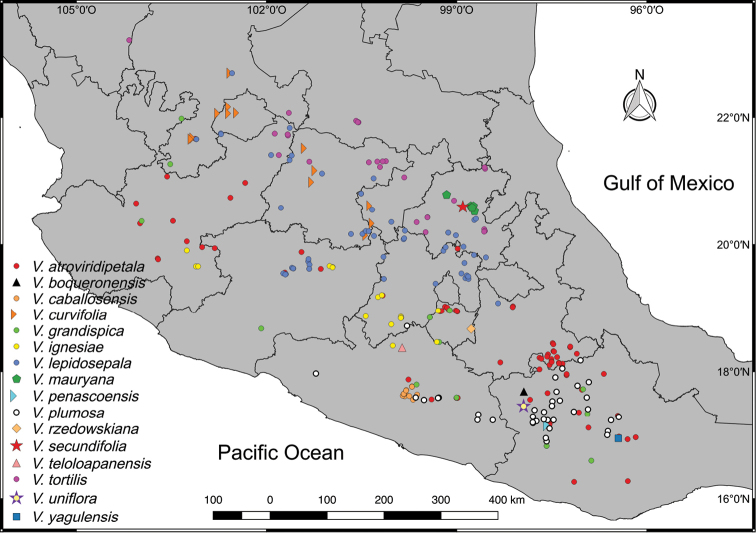
Geographical distribution of the genus *Viridantha*.

*Viridantha secundifolia* is only known from the state of Hidalgo in the western and eastern regions of Metztitlán and Tolantongo municipalities, respectively, where it grows on vertical walls in xerophilous scrubs at elevations between 1100 and 1900 m a.s.l. (Figs 1, 2).

#### Phenology.

Blooming in January and February.

#### Observations.

*Tillandsia mauryana* was described by Lyman B. Smith (1937), based on specimens from the canyon of Metztitlán, in the state of Hidalgo, Mexico. Espejo-Serna (2002) transferred the species to *Viridantha*. Ehlers (2009) described T. mauryana L. B. Sm. forma secundifolia, differentiating it from the typical form only by the secund disposition of its leaves. Besides, Ehlers (2009) mentioned that its populations grow separated from those of *T. mauryana*. The detailed analysis of the morphological characteristics of living and dried specimens of T. mauryana forma mauryana and T. mauryana forma secundifolia allowed us to detect that, in addition to the characteristic mentioned by Ehlers (2009), there are other differences in the plants of both populations such as: the shape of the rosettes (falcate vs. spherical); the length (1–1.2 cm vs. 1.5–2.5 cm) and the shape (broadly oblong to square vs. broadly elliptic) of the leaf sheaths; the length of the floral bracts (1.8–2 cm vs. 1–1.5 cm), the presence or not of a keel on the floral bracts (absent or visible only in the apex vs. present along the bract); and the colour of the anthers (pale to dark green vs. black).

*Viridantha grandispica* (Ehlers) Hern.-Cárdenas, Espejo & López-Ferr., *V. rzedowskiana* Hern.-Cárdenas, Espejo & López-Ferr. and *V. teloloapanensis* (Ehlers & Lautner) Hern.-Cárdenas, Espejo & López-Ferr., are other species morphologically similar to *V. secundifolia* (Table 1). *Viridantha secundifolia* differs from *V. grandispica* in the shape of the rosettes (falcate vs. spherical); in the shape of the leaf sheaths (broadly oblong to square vs. narrowly oblong); in the presence or not of a keel on the floral bract (absent or visible only in the apex vs. present along the bract); and in the shape of the sepals (lanceolate vs. ovate). *Viridantha secundifolia* differs from *V. rzedowskiana* in the shape of the rosettes (falcate vs. spherical); in the size of the leaf sheaths (1–1.2 cm × 0.8–1.1 cm vs. 1.8–2 cm × 1.5–1.7 cm); in the keel of the floral bract (absent or visible only in the apex vs. present along the bract); and in the shape of the sepals (lanceolate vs. ovate). *Viridantha secundifolia* differs from *V. teloloapanensis* in the shape of the rosettes (falcate vs. spherical); in the shape of the leaf sheaths (broadly oblong to square vs. ovate); in the number of the spikes (3–5 vs. 1); and in the shape of the sepals (lanceolate vs. ovate). So we concluded that T. mauryana forma secundifolia presents different and consistent morphological characteristics to those observed on the typical form and can be considered as a distinct species.

**Table 1.** Morphological differences amongst *Viridantha grandispica*, *V. mauryana*, *V. rzedowskiana*, *V. secundifolia* and *V. teloloapanensis*.

Characters	*V. grandispica*	*V. mauryana*	*V. rzedowskiana*	*V. secundifolia*	*V. teloloapanensis*
Rosettes shape in outline	Spherical	Spherical	Spherical	Falcate	Spherical
Leaf sheaths size (cm)	1–2 × 0.5–0.7	1.5–2.5 × 1–1.5	1.8–2 × 1.5–1.7	1–1.2 × 0.8–1.1	1–1.5 × 0.8–1.1
Leaf sheaths shape	Narrowly oblong	Broadly elliptic	Broadly oblong to square	Broadly oblong to square	Ovate
Leaf blades width (mm)	3–4	7–11	4.5–6	5–8	5
Floral bracts size (cm)	1.7–2.5 × 0.6–1	1–1.5 × 1–1.5	1.5–2 × 1–1.2	1.8–2 × 0.7–1.2	1–1.5 × 0.6
Floral bracts keel	Present	Present	Present	Absent or visible only in the apex	Present
Sepals width (mm)	4–6	3–5	4–6	2.5–3.5	3
Sepals shape	Ovate	Ovate	Ovate	Lanceolate	Narrowly elliptic
Anthers colour	Green	Black	Green	Pale to dark green	Green to black

#### Specimen examined.

MEXICO, Hidalgo: municipio de Metztitlán. 28 km sobre el camino que va de Metztitlán a Tolantongo (20°35'43"N, 98°54'09.9"W), 1103 m a.s.l., 3 February 2018, *R. Hernández-Cárdenas y A. Sarabia 2136* (UAMIZ).

### Viridantha uniflora

Taxon classificationPlantaePoalesBromeliaceae

Hern.-Cárdenas, Espejo & López-Ferr. sp. nov.

9552F6F3-9C73-5630-A26C-118F4ADD31E8

urn:lsid:ipni.org:names:60479385-2

Figs 2 , 3

#### Diagnosis.

*Viridantha uniflora* is similar to *V. boqueronensis* but differs in the shape (square vs. ovate to triangular) and the width of the leaf sheath (0.7–0.8 cm vs. 1 cm); the width (0.4–0.5 cm vs. 0.7–1.3 cm) of the spikes, the number of flowers per spike (always 1 vs. 2–5); and in the shape of the floral bract (ovate vs. elliptic).

#### Type.

MEXICO. Oaxaca: Distrito de Juxtlahuaca, municipio de Santos Reyes Tepejillo, en los alrededores del boquerón de Santos Reyes Tepejillo (17°26'58"N, 97°56'29"W), 1960 m a.s.l., 21 April 2018, *R. Hernández-Cárdenas*, *E. Negri & J. Conde 2156* (holotype: UAMIZ!; isotype: MEXU!).

#### Description.

Plants saxicolous, flowering 7–10 cm tall, 7–9 cm diameter; rosettes acaulescent, solitary or caespitose, falcate in outline. Leaves numerous, shorter or equalling the inflorescence; sheaths pale brown on both surfaces, nearly square, 0.8–1 cm long, 0.7–1 cm wide, glabrous towards the base on both surfaces; blades falcate, densely greyish lepidote, narrowly triangular, 3–6 cm long, 0.3–0.4 cm wide, apical portion long attenuate. Inflorescence pedunculated, falcate, one-branched, with 3–5 spikes; peduncle 2.5–3.5 cm long, 0.2–0.3 cm diameter, covered by the bracts of the peduncle; peduncle bracts similar to the leaves but reducing in size towards the apical portion, densely greyish lepidote; spikes green, erect and appressed, flattened, elliptic, 1.3–1.5 cm long, 0.4–0.5 cm wide; flowers erect and appressed, only one per spike; floral bracts green to green-brownish, ovate, 1–1.5 cm long, 0.5–0.6 cm wide, apex acute to acuminate, ecarinate to slightly carinate at the apex, glabrous adaxially, lepidote abaxially; sepals green, lanceolate, 1–1.3 cm long, 0.3–0.4 cm wide, apex acute, the two adaxial ones carinate, both surfaces glabrous or lepidote abaxially mainly on the keel; petals dark green, narrowly oblong, 1.5–1.8 cm long, 0.2–0.3 cm wide, apex rounded to obtuse; filaments white, 0.8–1.2 cm long, included; anthers pale green, 1.2–1.5 mm long; ovary green, ellipsoid, 2.5–3 mm long, 2–3 mm diameter; style white, 8–10 mm long; style branches green. Capsules not seen.

#### Habitat and ecology.

**Figure 3.**
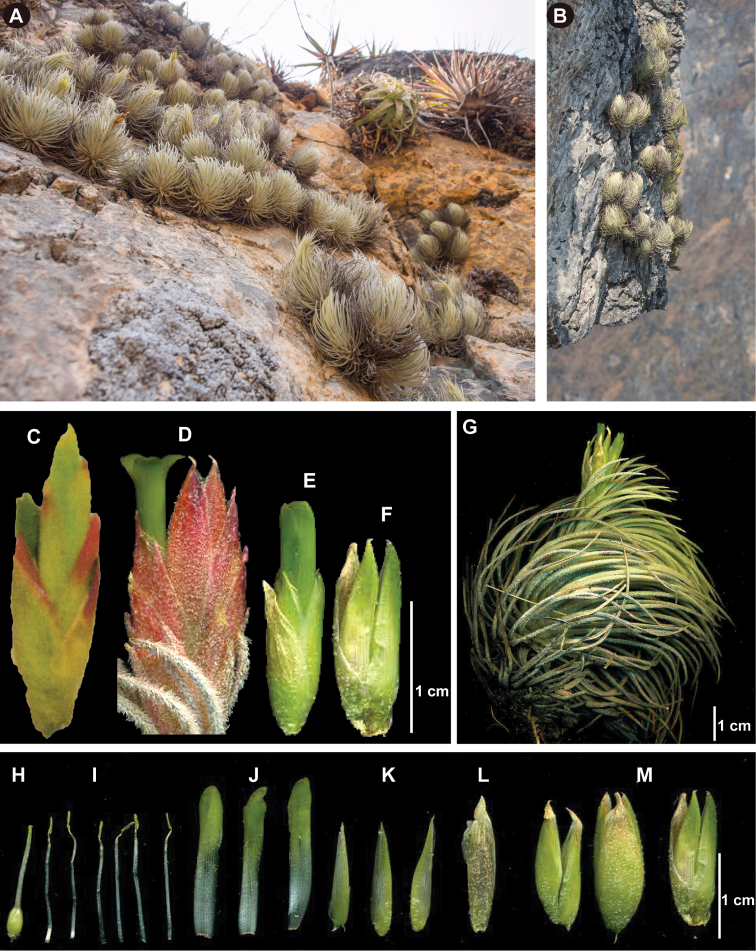
Morphological comparison between *Viridantha boqueronensis*, *V. penascoensis* and *V. uniflora* Hern.-Cárdenas, Espejo & López-Ferr. *V. uniflora***A–B** habit **E–F, M** spikes **G** plant with inflorescence **H** pistil **I** stamens **J** petals **K** sepals **L** floral bract (voucher: *Hernández-Cárdenas et al. 2156*, UAMIZ). *V. boqueronensis***C** spike (voucher: *K. and R. Ehlers EM7851*, MEXU). *V. penascoensis***D** spike (voucher: *Hernández-Cárdenas and Sarabia 2116*, UAMIZ). Photographs A–B by E. Negri Lavín; C–M by R. Hernández-Cárdenas.

*Viridantha uniflora* is only known from the boquerón of the Santos Reyes Tepejillo municipality, located in the Sierra Madre del Sur in the northwest region of the state of Oaxaca, where it grows on vertical walls in dry oak forests and tropical deciduous forests. The plants of *V. uniflora* grow in colonies, between 1700 and 1900 m a.s.l., on the cliffs of the boquerón amongst other saxicolous herbs. (Figs 2, 3).

#### Phenology.

The plants of *Viridantha uniflora* bloom in April and May.

#### Etymology.

The specific epithet refers to the presence of one flower per spike, condition only known in the proposed taxon.

#### Observations.

Plants of *Viridantha uniflora* had previously been collected by *J.I. Calzada 20057* (MEXU), but had been wrongly identified as *V. atroviridipetala* (Matuda) Espejo. However, *V. uniflora* differs from *V. atroviridipetala* in the outline shape of the rosettes (falcate vs. spherical); in the shape of the leaf sheaths (square vs. oblong to ovate); in the shape of the floral bracts (ovate vs. lanceolate to narrowly triangular) and in the number of flowers per spike (1 vs. 2–5). *Viridantha boqueronensis* and *V. penascoensis* grow in nearby locations to the type locality of *V. uniflora*, but without overlapping its distributions. These species share the saxicolous habit and the falcate rosettes in outline. However all these species are easily distinguishable from the newly proposed taxon (Table 2). *Viridantha uniflora* differs from *V. penascoensis* in the inflorescence (branched vs. simple); in the length and in the colour (green vs. red-pink) of the spikes (1.3–1.5 cm vs. 2–3 cm). *Viridantha uniflora* differs from *V. boqueronensis* in the shape of the leaf sheaths (square vs. ovate to triangular); in the number of flowers per spike (1 vs. 2–5); in the colour of the spikes (green vs. green with red-pink); in the shape of the floral bracts (ovate vs. elliptic); and in the presence or not of a keel on the floral bracts (absent or visible only in the apex vs. present along the bract.

**Table 2.** Morphological differences amongst *Viridantha boqueronensis*, *V. penascoensis* and *V. uniflora*.

**Characters**	***V. boqueronensis***	***V. penascoensis***	***V. uniflora***
Leaf sheaths size (cm)	0.9–1.4 × 0.8–1	0.6–1 × 0.7–0.8	0.8–1 × 0.7–0.8
Leaf sheaths shape	Ovate to triangular	Broadly ovate to square	Square
Leaf blades size (cm)	4–7 × 0.3–0.4	2.5–4 × 0.2–0.3	3–6 × 0.3–0.4
Spikes colour	Green with red	Red to pink	Green
Spikes number	5–7	1	3–5
Flowers number per spike	2–5	2–3	1
Spike size (cm)	1.5–3.5 × 0.7–1.3	2–3 × 0.8–1	1.3–1.5 × 0.4–0.5
Floral bracts size (cm)	1.2–1.7 × 0.5–0.8	1.5–2 × 0.5–1	1–1.5 × 0.5–0.6
Floral bracts shape	Elliptic	Ovate	Ovate
Floral bracts keel	Present	Absent or visible only in the apex	Absent or visible only in the apex
Sepals size (cm)	1–1.3 × 0.3–0.35	1.3–1.5 × 0.3–0.5	1–1.3 × 0.3–0.4
Sepals shape	Narrowly elliptic	Ovate	Lanceolate

#### Additional specimens examined (paratypes).

MEXICO, Oaxaca: Distrito Santiago Juxtlahuaca, municipio de Santos Reyes Tepejillo. 3 km al N de Santos Reyes Tepejillo rumbo a Corral de Piedra (17°27'N, 97°57'W), 1770 m a.s.l., 20 July 1995, *J. I. Calzada 20057* (MEXU); en los alrededores del boquerón de Santos Reyes Tepejillo (17°26'58"N, 97°56'29"W), 1960 m a.s.l., 18 March 2017, *R. Hernández-Cárdenas*, *F. Gómez y A. González 2120* (UAMIZ).

To facilitate the identification of the species of *Viridantha*, we include an artificial key for all representatives of the genus.

##### Key to the species of Viridantha

**Table:** 

1	Rosettes irregular or falcate in outline	**2**
–	Rosettes spherical in outline	**8**
2	Inflorescence branched	**3**
–	Inflorescence simple	**5**
3	Flower one per spike, spikes 1.3–1.5 cm long	***V. uniflora***
–	Flowers two or more per spike, spikes longer than 1.6 cm	**4**
4	Leaf blades 3–4 mm wide; floral bract elliptic	***V. boqueronensis***
–	Leaf blades 5–8 mm wide; floral bract ovate	***V. secundifolia***
5	Rosettes falcate, blades falcate	**6**
–	Rosettes irregular, blades squarrose	**7**
6	Plants longer than 5.1 cm; spikes terete	***V. curvifolia***
–	Plants shorter than 5 cm; spikes elliptic, flattened	***V. penascoensis***
7	Leaf sheaths broadly ovate to oblong; peduncle (in anthesis) longer than 4.1 cm and lesser than 2 mm diameter	***V. tortilis***
–	Leaf sheaths broadly oblong to square; peduncle (in anthesis) shorter than 4 cm and larger than 4 mm diameter	***V. lepidosepala***
8	Inflorescence conspicuously pedunculate, peduncle longer than 4.1 cm	**9**
–	Inflorescence sessile or peduncle shorter than 3.5 cm	**11**
9	Inflorescence simple, longer than 3.1 cm, flattened to terete, rarely with two small lateral spikes	***V. ignesiae***
–	Inflorescence branched, shorter than 3 cm long, flattened	**10**
10	Leaf blades wider than 2.1 mm; floral bracts triangular to ovate, carinate	***V. plumosa***
–	Leaf blades narrower than 2 mm; floral bracts elliptic to oblong, ecarinate to carinate only at the apex	***V. caballosensis***
11	Leaf blades wider than 7.1 mm; anthers black	***V. mauryana***
–	Leaf blades narrower than 7 mm; anthers green	**12**
12	Plants short caulescent; floral bracts elliptic	***V. yagulensis***
–	Plants acaulescent; floral bracts variable in shape but never elliptic	**13**
13	Spikes longer than 3.1 cm and wider than 1.1 cm	**14**
–	Spikes shorter than 3 cm and narrower than 1 cm	**15**
14	Leaf sheaths broadly ovate to square, blades wider than 4.1 mm; sepals shorter than 1.5 cm	***V. rzedowskiana***
–	Leaf sheaths narrowly oblong, blades narrower than 4 mm; sepals longer than 1.6 cm	***V. grandispica***
15	Spikes 3 or less; floral bracts shorter than 1.5 cm; sepals lanceolate	***V. teloloapanensis***
–	Spikes 4 or more; floral bracts longer than 1.6 cm; sepals narrowly elliptic	***V. atroviridipetala***

## Supplementary Material

XML Treatment for Viridantha secundifolia

XML Treatment for Viridantha uniflora

## Appendix 1

Examined specimens.

***Viridantha boqueronensis*** (Ehlers) Hern.-Cárdenas, Espejo & López-Ferr. Oaxaca: *Calzada 18325* (MEXU); *K. and R. Ehlers EM7851* (MEXU); *Lautner 92/57* (WU).

***Viridantha grandispica*** (Ehlers) Hern.-Cárdenas, Espejo & López-Ferr. Guerrero: *Ehlers EM040901* (WU); *Ehlers EM991902* (WU); *R. and K. Ehlers EM911305* (WU); *Franco 8* (FCME); *Limón 6* (FCME). Jalisco: *Flores et al. 1810* (CHAPA, IBUG, IEB); *Flores 2310* (CHAPA); *Guerrero et al*. *115* (IBUG). Michoacán: *Ehlers EM902503* (WU); *Steinmann 5156* (IEB). Morelos: *Ceja et al. 1049* (UAMIZ); *Flores-Palacios* and *Vergara 1048* (UAMIZ); *Hernández-Cárdenas and Moreno 2075* (UAMIZ); *Hernández-Cárdenas and Sarabia 2093* (UAMIZ); *López-Ferrari et al. 2865* (IEB, UAMIZ). Oaxaca: *Ceja et al. 17*62 (IEB, UAMIZ); *Espejo et al. 6492* (UAMIZ); *Ehlers EM030203* (WU); *Ehlers EM991204* (WU); *López-Ferrari et al. 3373* (UAMIZ); *Mendoza 1399* (UAMIZ); *Téllez et al. 16039* (FCME). Zacatecas: *Espejo et al. 7065bis* (UAMIZ); *Ehlers EM001405* (WU); *Ramírez-Díaz et al. 184* (IBUG).

***Viridantha mauryana*** (L.B. Sm.) Espejo. Hidalgo: *Ceja et al. 1967* (UAMIZ); *Ceja et al. 1768* (UAMIZ); *Gómez 533* (IEB); *Gold 2* (MEXU); *Hernández-Cárdenas et al. 2090* (UAMIZ); *López-Ferrari et al. 2133* (UAMIZ); *Maury 5747* (GH).

***Viridantha penascoensis*** (Ehlers & Lautner) Espejo & López-Ferr. Oaxaca: *Hernández-Cárdenas* and *A. Sarabia 2116*, (UAMIZ); *Ehlers EM030202* (MEXU).

***Viridantha rzedowskiana*** Hern.-Cárdenas, Espejo & López-Ferr. Morelos: *Hernández-Cárdenas et al. 2108* (UAMIZ).

***Viridantha teloloapanensis*** (Ehlers & Lautner) Hern.-Cárdenas, Espejo & López-Ferr. Guerrero: *Lautner et al. EM060902* (MEXU, WU); *Schatzl 80/7* (WU).

***Viridantha uniflora*** Hern.-Cárdenas, Espejo & López-Ferr. Oaxaca: *Calzada 20057* (MEXU); *Hernández-Cárdenas et al. 2156* (UAMIZ); *Hernández-Cárdenas et al. 2120* (UAMIZ).
